# The Investigation of the Association of Cutaneous Leishmaniasis in Biopsy Specimens of the Patients with Granulomatous Disease and Skin Cancer Using the Molecular Method

**DOI:** 10.18502/ijpa.v15i3.4194

**Published:** 2020

**Authors:** Gülnaz ÇULHA, Asena Çiğdem DOĞRAMACI, Sibel HAKVERDİ, İlke Evrim SEÇİNTİ, Özkan ASLANTAŞ, Ebru ÇELİK, Tuğba KAYA

**Affiliations:** 1. Department of Parasitology, School of Medicine, University of Hatay Mustafa Kemal, Hatay, Turkey; 2. Department of Dermatology, School of Medicine, University of Hatay Mustafa Kemal, Hatay, Turkey; 3. Department of Medical Pathology, School of Medicine, University of Hatay Mustafa Kemal, Hatay, Turkey; 4. Department of Microbiology, School of Veterinary Science, University of Hatay Mustafa Kemal, Hatay, Turkey

**Keywords:** Cutaneous leishmaniasis, Granulomatous diseases, Skin cancer

## Abstract

**Background:**

Clinically, cutaneous leishmaniasis (CL) can be confused with granulomatous diseases and skin cancers, and it may lead to erroneous diagnosis and treatment. Diagnosis based and histopathology can have some difficulties due to low number of parasites, especially in chronic CL cases. We aimed to emphasize the necessity of considering CL in the differential diagnosis for cases of granulomatous diseases and basal cell carcinoma, particularly in areas where CL is endemic.

**Methods:**

One hundred and seven paraffin-embedded tissue biopsy specimens were selected from the archive, as of 2002, of Pathology Department, School of Medicine, University of Hatay Mustafa Kemal in Hatay, Turkey. After DNA isolation, performed with the samples were used for PCR analysis with specific 13A, 13B primers targeting kinetoplastid DNA (kDNA) found in all *Leishmania* species. Another PCR was performed with LITSR and L5.8S primers targeting ITS-1 internal-transcribed-spacer-1 (ITS-1) region to subtype positive samples. Then these samples were further analyzed for subtyping with PCR-RFLP using HaeIII enzyme (BsuRI).

**Results:**

Ten out of 107 tissue specimens were positive via kDNA-PCR. Lupus vulgaris, sarcoidosis, skin lymphoma and *Leishmania* cutis appeared in 9 out of 10 positive specimens. One of the cases presented with a mass on the cheek and was pre-diagnosed with hemangioma, but leishmaniasis did not appear. All of 10 specimens were diagnosed as granulomatous dermatitis. Two out of 10 samples, found positive with kDNA-PCR, were analyzed with ITS-1-PCR and identified as *L. infantum/donovani* after RFLP.

**Conclusion:**

Molecular methods should be utilized in the differential diagnosis of CL to eliminate false diagnoses of granulomatous diseases and skin cancers.

## Introduction

Leishmaniasis is caused by intracellular parasites of the genus *Leishmania* and transmitted by *Phlebotomus* vector. It generally appears in three clinical forms: visceral leishmaniasis (VL), cutaneous leishmaniasis (CL) and mucocutaneous leishmaniasis (MCL). According to WHO, approximately 1.5 million CL cases have been reported and more than 350 million people were under risk (1, 2, 3).

Although *L. tropica* and *L. major* species are the most common reason of CL in Turkey, previous studies showed that *L infantum* causes CL as well (4, 5).

CL has similar clinical symptoms with several skin diseases such as pseudolymphoma, sarcoidosis, eczema, skin tuberculosis, anthrax, myiasis, syphilis, bacterial skin infections, malignant ulcers (such as squamous cell carcinoma) which can result in erroneous diagnoses. Moreover, the possible risk of developing basal cell carcinoma on the scar surface is reported (6).

The diagnosis of CL is based on the microscopic observation of the amastigotes within the dermal scrap specimen. The diagnosis can be difficult in the regions where CL is not endemic and with the cases that are lack of experienced staff. It is required to use advanced diagnostic methods such as histopathological examination, culture and molecular methods to analyze skin lesion biopsies, when the parasite cannot be detected in the smear. The low number of parasites in the histopathological examination, particularly in chronic cases, may increase the margin of error (7).

In the histopathological examination, lupus vulgaris, leprosy, acne rosacea, and sarcoidosis should be considered in the differential diagnosis of CL. There can be difficulties in the diagnosis of CL especially when organisms are at low number in granulomatous infiltrate. The presence of perineural infiltration in leprosy, caseification in lupus vulgaris, and schumann bodies in sarcoidosis allows the differentiation of CL from other granulomatous diseases (8).

CL can directly or indirectly change the diagnosis and the course of malignancies. Besides, CL is an important factor that paves the way for skin cancers. Therefore, this study was planned with the aim of emphasizing that due to the difficulties in the accurate diagnosis of CL among its various similar clinical forms the differential diagnosis should be considered by excluding atypical granulomatous diseases (paracoccidioidomycosis, sporotrichosis, chromomycosis, cutaneous tuberculosis, lupus vulgaris) and basal cell carcinomas, particularly in the regions where CL is endemic.

## Materials and Methods

The study involved tissue biopsy specimens with granulomatous agents such as granulomatous dermatitis, basal carcinoma, squamous carcinoma, sarcoidosis, and tuberculosis collected from the tissue archive, as of 2002, sent by various clinics to the Pathology Department of Hatay Mustafa Kemal University, Health Practices and Research Hospital, Hatay, Turkey. The clinical diagnoses before and after biopsy about the formalin-fixed and paraffin-embedded tissue samples were evaluated. All of the forms included the patients information such as age, gender etc.

### Ethical statement

Ethical approval for the present study was obtained from the Clinical Research Ethics Committee of Tayfur Ata Sökmen Faculty of Medicine, Hatay Mustafa Kemal University (Research Code: 2018/59).

### Histopathology and Histochemistry

Overall, 107 biopsy samples were stained with hematoxylin & eosin (H&E) and Giemsa in Pathology department. When *Leishmania* amastigotes were not detected, they were considered as negative biopsy sample. The clinical diagnosıs before biopsy, the tissue samples were evaluated histopathologically such as granulomatous inflammation, sarcodial, basal and squamous carcinoma. Then paraffin embedded tissue blocks of patients conserved in pathology department archive.

### DNA extraction from the paraffin-embedded tissue and PCR

A total of 107 paraffin-embedded tissue blocks of patients referred to pathology from various clinics were cut into 5–10 μm slices. Then, the tissue biopsy specimens of paraffin-embedded were transferred in 10% formalin. After paraffin was scraped off the sections with the help of a sterile scalpel without damaging the biopsy tissue, the samples were washed with xylene for 45 min, then with alcohol, and finally with distilled water. The samples were kept in a lysis buffer (10% Sodium Dodecyl Sulfate, 1M NaCl, 10 mg/ml proteinase K, Tris EDTA) overnight to dissolve the biopsy specimens. DNA isolation from the tissue homogenates was made by using a commercial extraction kit (QIAamp DNA Mini Kit; Qiagen, Hilden, Germany). The DNA samples were stored at −20 °C until it was used.

The isolated DNA samples were used to amplify kinetoplast DNA (kDNA) found in *Leishmania* species by using PCR method with the following primers 13A: 5′-dGTGGGGGAGGGGCGTTCT-3′ and 13B: 5′dATTTTACACCAACCCCCAGAGT-3′. The amplicon size of the PCR reaction, as of *Leishmania* kDNA, was 116 base pairs (bp) (9). KDNA-positive samples were used to perform another PCR for subclassification of *Leishmania* species. For this purpose, the ITS-1 gene region was amplified with primers; LITSR: 5′-CTGGATCATTTTCCGATG-3′ and L5.8S: 5′-TGATACCACCACTTATCGCACTT-3′ which is ensued by a RFLP analysis (10, 11). For PCR reactions, 5 μl template DNA, 0.5 μl of each primer, 0.25 μl Taq DNA polymerase, 1 μl dNTP mix, 5 μl 10x Taq polymerase buffer, and 4 μl MgCl_2_ buffer was mixed and filled up to 50 μl with distilled water. Then PCR reaction were placed in a thermal cycler with the following PCR cycling conditions: initial denaturation for 7 min at 94 °C, 35 cycles of 40 sec at 94 °C, 40 sec at 56 °C, and 40 sec at 72 °C, and 7 min at 72 °C. 1.5% agarose gel was prepared and the PCR samples were run at 80 volts for 1 hour and visualized under ultraviolet light for further analysis. Positive and negative controls were used in each experiment to eliminate possible false positive signals. *L. infantum/donovani, L. tropica, and L. major* were used as reference strains which are found in Hatay province located in the south region of Turkey.

### PCR-RFLP

The PCR amplicons that were amplified with LITSR and L5.8S primers were used for RFLP analysis. The final reaction volume was 20 μL containing; 5μL PCR product, 2 μL BsuRI enzyme, and 2 μl restriction buffer. The reaction mixture was incubated at 37 °C overnight and visualized after running on 3% agarose gel for 2 hours. The samples were subtyped by comparing the signals based on reference strains (*L. tropica, L. major, L. infantum/donovani*).

## Results

The clinical data of 10 patients included in the current study could not be attained from the polyclinic archive. The rest of 97 tissue specimens belonged to 56 males and 41 females of which consisted of 75 Turkish, and 21 Syrian patients. Age distribution ranged between 6 and 97. The patients were from mainly the districts of Hatay such as Altınözü, Antakya, Belen, İskenderun, Kırıkhan, Reyhanlı, Samandağ, and Yayladağ, also from Syrian regions such as Deirozzor, Halep, İdlip and other regions of Turkey such as Adana, Ankara, Kahramanmaraş, and Kilis. All of the patients were referred to pathology department from dermatology (22), otorhinolaryngology (7), pathology (1), cardiology (1), and plastic and reconstruction (66) polyclinics with various pre-diagnoses such as BCC, SCC, lupus vulgaris, dermatofibroma, sarcoidosis, leishmaniasis, Jessner lymphocytic infiltration, discoid lesion erythematosus, lichen planus, skin lymphoma, pseudolymphoma, and hemangioma.

**Fig. 1:**
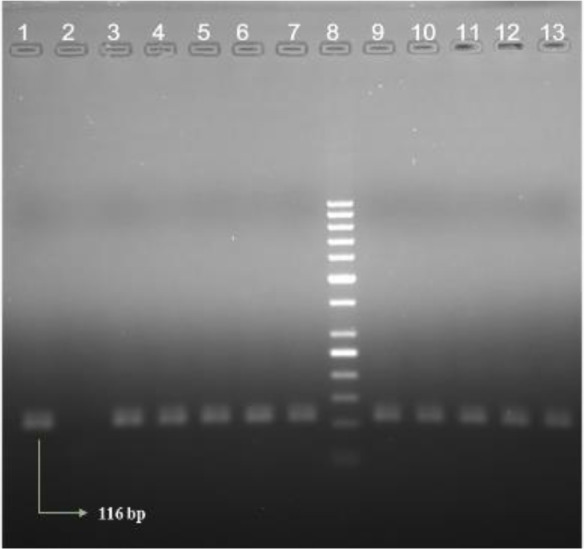
CL suspected samples using kDNA-PCR [1: PC (Positive control), 2: NC (Negative control), 3–7: Positive patient samples, 8: Marker (50bp), 9–13: Positive Patient samples

Ten of 107 tissue specimens were found positive with kDNA PCR. Lupus vulgaris, sarcoidosis, Bowen’s disease, tinea incognita, discoid lesion erythematous, skin lymphoma, and *Leishmania* cutis were included in the clinical pre-diagnosis of 9 out of 10 positive samples. One of the cases presented with a mass on the cheek and was pre-diagnosed as hemangioma, but not leishmaniasis. Ten samples in total had a diagnosis of Granulomatous Dermatitis. Lesion durations ranged from 10 months to 15 years. Lesion types were defined as nodular, ulcerated, plaque, erythematous, crusted ulcers, and massive. Lesions were mostly located in the head and upper extremity. According to the histopathological examination, granuloma of a large number of epitheloid histiocytes at various sizes under multilevel squamous epithelium which exhibited hyperkeratosis and inflammatory cell infiltration of dense lymphocytes and plasma cells was observed in six samples (Table 1) (Fig. 1).

**Table 1:** Main findings and general characteristics of samples found positive by kDNA-PCR

***Lesion Location***	***Age***	***Gender***	***Location***	***Poly-clinic***	***Lesion Duration***	***Type Of The Lesions***	***Differential Diagnosis***	***Histapathological And İmmunochemical Diagnosis***
Right hand	97	F	Belen / Hatay	D	2 year	Crusted ulcerated lesion	Lupus vulgaris / Lymphoid leishmaniasis / Sarcoidosis / Bowen’s disease / Tinea incognito	Granulomatous dermatitis
Right hand	65	F	Kırıkhan/ Hatay	D	10 month	Crusted lesion	Leishmania cutis / Sarcoidosis	Granulomatous dermatitis
Forehead	67	M	Aktepe-Hassa / Hatay	D	2 year	Plaques lesion	Discoid lesion / Sarcoidosis / Lymphoma	Granulomatous dermatitis
Brow	46	F	İskende-run / Hatay	D	15 year	Erythematous plaques	Leishmania cutis / Sarcoidosis / Discoid lupus Erythematous / Bowen’s diesease / Jessner’s lymphocytic infiltration	Granulomatous dermatitis
Forehead	45	M	Islahiye / Gaziantep	PS	4 year	Plaques	Leishmania cutis / Sarcoidosis / Skin lymphoma	Granulomatous dermatitis
Left arm	31	F	Kırıkhan / Hatay	PS	--	Nodule	Dermatofibriom / Leishmania cutis / Sarcoidosis / Jessner’s lymphocytic infiltrate	Granulomatous dermatitis
Right cheek	31	F	Kırıkhan / Hatay	PS	1 year	Mass	Haemangioma	Chronic Granulomatous dermatitis
Ear	20	F	Antakya / Hatay	D	-----	Erythematous - Squamous infiltrating lesion	Leishmaniasis / Lupus vulgaris / Deep tinea infection	Chronic Granulomatous dermatitis
Leg	29	M	Syria	D	1 year	Erythematous nodular lesion	Leishmania cutis / Lymphocytomatosis / Sarcoidosis / Lymphoma	Granulomatous dermatitis / In accordance with CL
Dorsal foot	82	F	Belen / Hatay	D	----	Erythematous ulcerative lesion	Leishmania cutis / Pyoderma / Gangrenosum / Cutaneous tuberculosis / SCC / Skin fungi	Granulomatous dermatitis

**Fig. 2:**
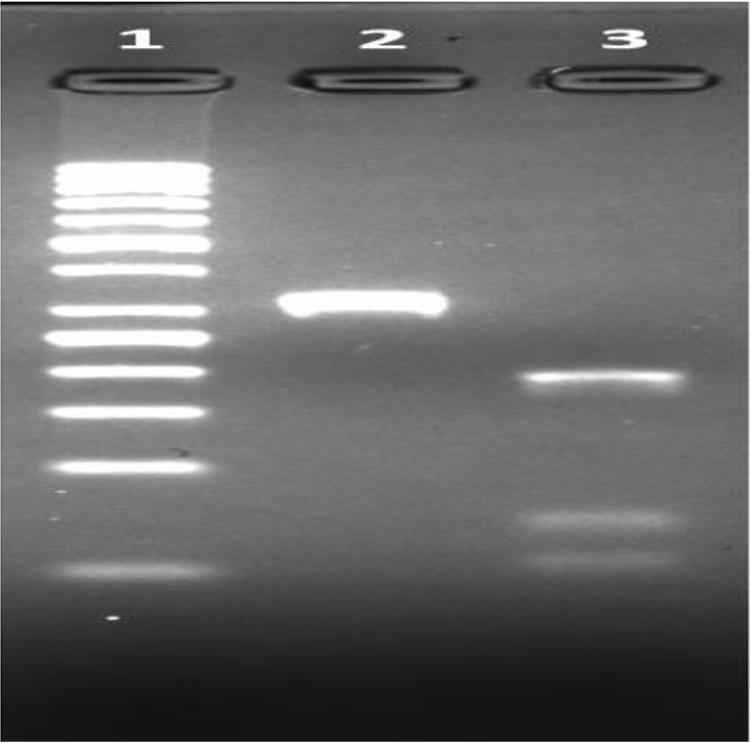
ITS-1 RCR-RFLP [1: Marker (50 bp), 2: Positive patient sample (ITS-1 PCR product), 3: Positive patient sample (*L. infantum/donovani*)]

**Fig. 3:**
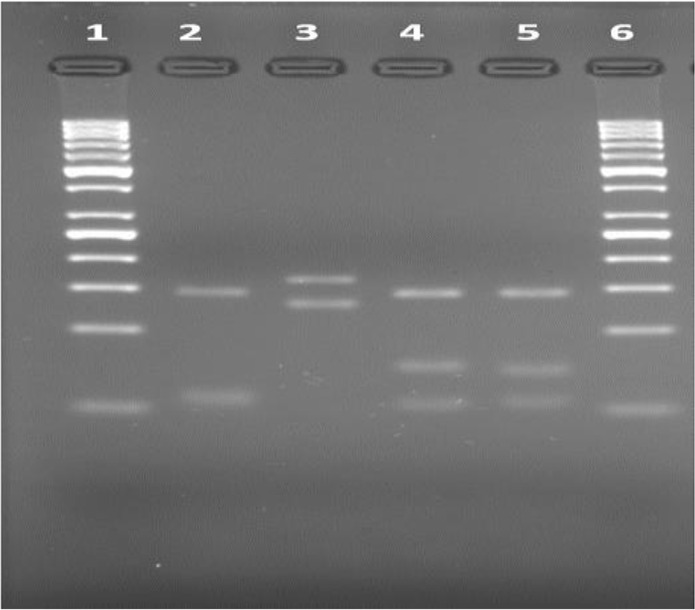
ITS-1 RCR-RFLP [1: Marker (50 bp), 2: RF1 (*L.tropica*), 3: RF2 (*L.major*), 4: RF3 (*L.infantum/donovani*), 5: Positive Patient sample (*L.infantum/donovani*), 6: Marker (50 bp)]

Ten positive samples were studied with ITS-1 PCR and only two samples were detected as positive due to the low amount of DNA, which were subtyped as *L. infantum/donovani with* PCR-RFLP (Fig. 2, 3).

## Discussion

CL lesions may lead to the formation of skin collapse scars in untreated cases although they usually tend to heal spontaneously. It is known that scar tissue is associated with various skin cancers, especially basal cell carcinoma. Therefore, considering the risk of skin cancer development, the significance of CL’s pre-diagnosis and treatment increases. CL might be confused with several granulomatous diseases, therefore particularly in chronic CL cases, the differential diagnosis is required to exclude basal and squamous cell carcinomas. In 1988, Suster and Ronnen reported a basal cell carcinoma which emerged from *Leishmania* scar 3 years after the lesion’s treatment (12). Furthermore, in 2003, a squamous cell carcinoma which developed in a *Leishmania* scar 59 years after the treatment was reported (13).

Generally a period of 35 years must elapse before the appearance of a malignant lesion on chronic scar. A patient with leishmaniasis developed basal cell carcinoma 30 years after the first diagnosis (14). The atrophy of adnexal structure in scar areas could make affected tissues more susceptible to the impacts of ultraviolet radiation and other exogenous carcinogens, and the exposure of injured sites to radiation could trigger malignant transformation (14).

Chronic CL cases might not be seen in smears and histochemical touch preparations due to secondary infection, negligence, wrong treatment, the decrease in parasite load, etc. Molecular methods are needed in this case.

In a previous study, 130 biopsy specimens with chronic lesion, which were defined as granulomatous dermatitis and estimated as negative in smears, were studied with PCR using *Leishmania*-specific primers. Besides, 27 of 130 biopsy specimens were evaluated as positive. Only 3 of these positive samples were assessed as clinically positive for CL (15).

In a study comparing diagnostic methods with 25 tissue samples, the positivity ratios of microscopic examination, culture method, and kDNA-PCR were 44%, 68%, and %100 respectively (9). Moreover Akkafa et al, 49 (96%) of 51 anthroponotic CL suspected patients were determined as positive with PCR (16). In another study, with comparing microscopy, culture and kDNA-PCR, in suspected CL cases were detected positivity 29 (53%), 34 (62%), and 30 (55%), respectively (17). Negative Giemsa stained smears of 81 suspected CL patients showed nine (11,1%) positive with kDNA-PCR (18). In our study, 10 samples (9,4%) were positive after analysis with kDNA-PCR.

Akçalı et al, observed granulomatous infiltrations involving *Leishmania* parasites in a patient with clinical pre-diagnosis of squamous cell carcinoma and confirmed the diagnosis of leishmaniasis with PCR. They underlined that CL should be considered in the patients who are particularly from endemic regions and have nonhealing or unusual dermatological lesions since CL could be imitated several other skin diseases (19). Hatay is an endemic province in terms of CL (20). Analyzing the clinical pre-diagnosis of all tissue samples, it was reported that mostly the patients from endemic rural regions apply to polyclinics and the majority of lesions were in the face and upper extremities. Lupus vulgaris and sarcoidosis were placed on the top along with CL in all biopsy samples with the pre-diagnosis of CL in our study.

Among 40 people with skin diseases other than CL, parasites could not be shown in-vitro histopathological or culture methods (21). According to kDNA-PCR data in the same study, tissue biopsy specimens of 7 cases with skin lesions were positive for leishmaniasis, although all samples were clinically suggestive of CL. As a consequence, they notified that PCR performed on DNA extracted from formalin-fixed and paraffin-embedded tissue samples was a valuable method in terms of CL diagnosis in chronic cases especially with low parasite load in the lesion (21). Likewise, in our research, 10 samples were positive after PCR-based DNA analysis of 107 paraffin-embedded tissue samples.

In a study conducted to evaluate the validity of the PCR method in CL diagnosis, kDNAPCR was performed on paraffin-embedded skin biopsies and 52 of 54 samples were positive for *Leishmania* (22). Shirian et al compared 100 paraffin-embedded CL-suspicious samples with conventional, molecular, and immunohistochemical methods and found that kDNA PCR results demonstrated the highest sensitivity with the rate of %100 (23).

In a case study a patient with chronic lesion on the face was directed to histopathological examination after pre-diagnosis of squamous cell carcinoma, and eventually diagnosed with *Leishmaniasis*. The patient got recovered after treated with miltefosine (24). In our study, positive three patients who applied to plastic surgery with plaques, nodule, mass of lesion type were found leishmaniasis with kDNA-PCR.

In study concerning a patient with the history of substance abuse and chronic obstructive pulmonary disease who had multiple papular, crusted, and severe ulcerative lesions, leishmaniasis was suspected as a result of smear and skin biopsy, yet ITS-1 PCR was performed for a definitive diagnosis. In the same study PCR-RFLP results indicated the presence of *L. major*. So it was pointed out that in countries where CL was endemic, the atypical form of CL should be paid more attention (25).

In Turkey, leishmaniasis showed in a patient who was first diagnosed with perichondritis and treated, and then pre-diagnosed with anthrax (26). In another case study, conducted on a 22-year-old female pregnant patient carrying lesion in the ear amastigotes of *Leishmania* parasite was detected (27). In Syria, for a 67-year-old male patient with a case in the ear for 6 months, amastigotes of *Leishmania spp* was observed in hematoxylin-stained preparations (28).

Clinical data of 10 patients among the collected tissue samples in our study could not be attained. The subjects we used in the current study consisted of 6 to 97-year-old 56 males and 41 females. The majority of the patients were from endemic districts of Hatay such as Altınözü, Antakya, Belen, İskenderun, Kırıkhan, Reyhanlı, Samandağ, and Yayladağı, also from the cities in Syria such as Deir ez-Zor, Aleppo, and Idlib, as well as from different provinces in our country such as Adana, Ankara, Kahramanmaraş, and Kilis. Most of the patients were referred to pathology unit of our hospital from plastic surgery and dermatology polyclinics with various pre-diagnoses such as BCC, SCC, lupus vulgaris, dermatofibroma, sarcoidosis, leishmaniasis, jessner lymphocytic infiltration, lichen planus, skin lymphoma, pseudolymphoma and hemangioma.

In our study, biopsy material was taken from a patient who had “hemangioma” on the face. This patient was not pre-diagnosed as *Leishmania* although the patient had that lesion for more than 1 year. In parallel, another patient who applied to plastic surgery clinic complained about “a mass in the left arm elbow inferior”, and pre-diagnosed as “Dermatofibroma, sarcoidosis, leishmaniasis, jessner lymphocytic infiltration”. The diagnosis of the patient was “noncaseating granulomatous dermatitis” based on the pathological report of tissue biopsy. Although leishmaniasis, sarcoidosis, acne vulgaris, tuberculosis were reported in the pre-diagnoses of other tissue samples, *Leishmania* parasite could not be observed in tissue biopsy materials except one sample. Nevertheless, based on our observations, the necessity of clinical examination in terms of leishmaniasis was emphasized.

Despite CL as endemic in our country, leishmaniasis can be overlooked in the patients who applied to different clinics. Additionally, the patient samples considered as chronic and suspicious in particular should be further analyzed by using the molecular method to avoid the misdiagnosis of CL cases.

## Conclusion

CL is a disease with wide spectrum of clinical symptoms that may cause wrong treatments and/or surgical interventions since it can imitate many skin diseases. Thus CL can be overlooked as a result of misdiagnoses. Especially due to the increasing number of immigrants in Turkey, exclusion of CL in all granulomatous diseases, particularly in long-term chronic cases, such as basal and squamous carcinoma and sarcoidosis, the provision of more information for health institutions, and cooperation between laboratories and clinics gain high significance.
